# Network-based analysis of omics data: the LEAN method

**DOI:** 10.1093/bioinformatics/btw676

**Published:** 2016-12-06

**Authors:** Frederik Gwinner, Gwénola Boulday, Claire Vandiedonck, Minh Arnould, Cécile Cardoso, Iryna Nikolayeva, Oriol Guitart-Pla, Cécile V Denis, Olivier D Christophe, Johann Beghain, Elisabeth Tournier-Lasserve, Benno Schwikowski

**Affiliations:** 1 Univ Paris Diderot, Sorbonne Paris Cité, UMRS 1161, Paris, France; 2 INSERM, U1161, Paris, France; 3 Univ Paris Diderot, Sorbonne Paris Cité, UMRS 958, Paris, France; 4 INSERM, U958, Paris, France; 5 Systems Biology Lab, C3BI, USR 3756, Institut Pasteur/CNRS, Institut Pasteur, Paris, France; 6 Functional Genetics of Infectious Diseases Unit, Institut Pasteur, Paris, France; 7 Univ Paris-Descartes, Sorbonne Paris Cité, Paris, France; 8 Unité 1176, INSERM, Univ Paris-Sud, Université Paris-Saclay, Le Kremlin-Bicêtre, France; 9 Genetics and Genomics of Insect Vectors, Institut Pasteur, Paris, France; 10 AP-HP, Groupe Hospitalier Saint-Louis Lariboisière-Fernand-Widal, Paris, France

## Abstract

**Motivation:**

Most computational approaches for the analysis of omics data in the context of interaction networks have very long running times, provide single or partial, often heuristic, solutions and/or contain user-tuneable parameters.

**Results:**

We introduce local enrichment analysis (LEAN) for the identification of dysregulated subnetworks from genome-wide omics datasets. By substituting the common subnetwork model with a simpler *local* subnetwork model, LEAN allows exact, parameter-free, efficient and exhaustive identification of local subnetworks that are statistically dysregulated, and directly implicates single genes for follow-up experiments.

Evaluation on simulated and biological data suggests that LEAN generally detects dysregulated subnetworks better, and reflects biological similarity between experiments more clearly than standard approaches. A strong signal for the local subnetwork around Von Willebrand Factor (VWF), a gene which showed no change on the mRNA level, was identified by LEAN in transcriptome data in the context of the genetic disease Cerebral Cavernous Malformations (CCM). This signal was experimentally found to correspond to an unexpected strong cellular effect on the VWF protein. LEAN can be used to pinpoint statistically significant local subnetworks in any genome-scale dataset.

**Availability and Implementation:**

The R-package *LEANR* implementing LEAN is supplied as supplementary material and available on CRAN (https://cran.r-project.org).

**Supplementary information:**

Supplementary data are available at *Bioinformatics* online.

## 1 Introduction

The organization of the molecular machinery of cells is thought to be inherently modular (Alon, 2003; Hartwell *et al.*, 1999). When studying large-scale datasets, once gene-level scores have been computed, a common next step is thus to aggregate them to the level of *gene sets*.

Pathway analysis focuses on enrichment in annotated gene sets, such as genes involved in a common biological process. For the remainder of this article, we will use the terms *pathway* and *gene set* interchangeably in the above sense of a set of genes sharing a common functional annotation. Significant scores for a particular pathway suggest specific higher-level functional interpretations of the dataset (Khatri *et al.*, 2012). The MSigDB database (Subramanian *et al.*, 2005) for example includes pathways corresponding to genes that share functional annotations, chromosomal locations or cis-regulatory motifs, or are part of specific molecular (e.g. oncogenic or immunologic) signatures.

Subnetwork-based analyses (for a recent review see (Mitra *et al.*, 2013)) follow the same general approach, except that candidate gene sets are subnetworks of physical or functional interaction networks that are interconnected by known interactions. An obvious advantage relative to pathway-based approaches is that gene sets representing novel biological functions can be discovered. However, the number of candidate subnetworks to be considered is usually astronomical, and only identifying the most significant subnetwork is computationally hard, even for simple versions of the problem (Ideker *et al.*, 2002). As a result, existing methods resort to computationally intensive heuristics (Ideker *et al.*, 2002), to solving limited versions of the computational problem (Backes *et al.*, 2012) and/or entirely different problem formalizations (West *et al.*, 2013). As a consequence of the computational hardness of the subnetwork problem, current methods do not allow exhaustive evaluation of all possible subnetworks and often require the user to set additional parameters, a non-trivial step that can strongly influence the methods’ results in often unclear ways. Furthermore, the employed subnetwork scores of widely used methods have been shown to be biased with respect to subnetwork size (Nacu *et al.*, 2007; Rajagopalan and Agarwal, 2005) and most methods do not provide a sound estimate of statistical significance of their solutions. Another typical challenge is the biological interpretation of resulting subnetworks. Firstly, the resulting subnetworks have typically not been studied previously, and interpretation and hypothesis generation from the observed results can therefore not rely upon existing knowledge. Secondly, experimental methods typically do not allow the study of subnetworks in their entirety, so that a non-trivial prioritization step is required before further validation.

As a consequence, comparative studies on medically relevant data have reported poor consistency between individual methods (Jiang and Gribskov, 2014) and questioned the merit of applying subnetwork-based methods altogether (Staiger *et al.*, 2013)

We present here a novel network-based approach, termed *local enrichment analysis* (*LEAN*). LEAN is designed to avoid computational and statistical issues through the use of a strongly constrained subnetwork model. The strong constraint permits a combination of fundamental advantages, relative to existing network analysis approaches. In particular, the underlying optimization problem is efficiently solvable, allows to survey all solutions (not just the optimal one), it has no parameters that have to be tweaked by the user, and LEAN subnetworks of interest imply specific genes for experimental follow-up. Supplementary table S1 compares key features of LEAN with a range of other subnetwork analysis approaches.

We evaluated the performance of LEAN by comparing it to previously published subnetwork detection methods on simulated pathways and showed that it is able to extract biologically meaningful common pathways even on a relatively small number of publicly available datasets. An application to a transcriptomic dataset of the response to invalidation of CCM genes in mouse models led to the discovery of the previously unknown involvement of Von Willebrand Factor (VWF) in the pathophysiology of CCM disease.

## 2 Methods

In the following paragraphs, we will introduce the key concepts of the LEAN method. For better readability, detailed descriptions of how presented results were obtained are relegated to the Results and supplementary material, respectively.

### 2.1 The local subnetwork model

**Fig. 1:**
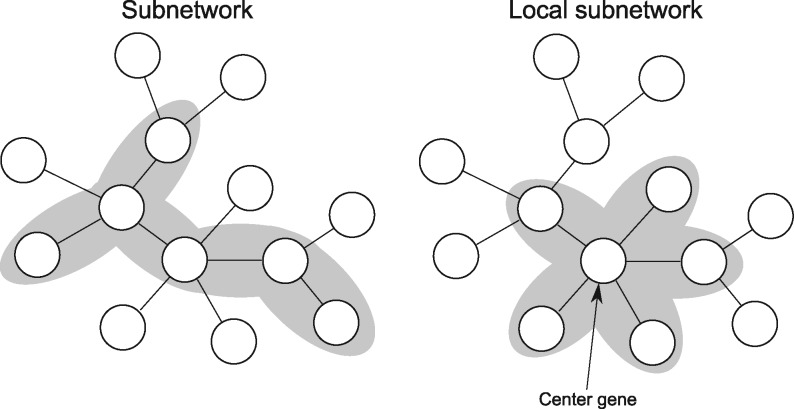
*Subnetwork* and *local subnetwork* pathway models. Local subnetworks are specific subnetworks that consist of a center gene and its direct network neighbors

We introduce here a novel network-based analysis approach integrating genome-wide measures of statistical significance (*P*-values) with large-scale interaction networks. It is based on a *local subnetwork model*, which assumes that higher-order biological activity can be detected by aggregating signals from a single gene and its direct network neighbors (cf. Fig. 1).

The local subnetwork model is much simpler than the common (unconstrained) subnetwork model, in terms of computational complexity, and the assessment of statistical significance. While the number of subnetworks is typically exponential in the number of genes, networks contain only a single local subnetwork per gene. The identification of optimal subnetworks is computationally NP-hard (Ideker *et al.*, 2002), whereas optimal local subnetworks can be identified by examining all genes and their neighborhoods in turn. The relatively low number of local subnetworks also allows the straightforward calculation of empirical *P*-values while for many subnetwork-based analysis methods, no procedures exist to calculate statistical significance.

### 2.2 Local enrichment analysis

LEAN is based on two ingredients: A list of measures of statistical significance (*P*-values) for some or all genes and an interaction network. In many applications, *P*-values originate from a statistical test for differential expression, such as *limma* (Smyth, 2004). While the approach is readily applicable to other types of datasets, we will describe it in the following using the example of its application to the results of a transcriptomic experiment evaluating differential gene expression (input *P*-values). Analysis is performed using the given interaction network restricted to genes for which an input *P*-value has been calculated based on the transcriptomic data. A *local subnetwork*$N_{g}$ consists of a subset of genes formed from a designated *center gene*g and its direct interactors in the given network. Candidate subnetworks are all local subnetworks $N_{g}$.

An equivalent characterization of local subnetworks uses the notion of graph *radius*. The *eccentricity* of a node *g* is the maximal graph distance of *g* to any other node *h*. A node *g* is a *graph center* if it has minimal eccentricity, which is also called radius. Using these notions, local subnetworks are exactly those subnetworks that have a radius of 1.

### 2.3 LEAN *P*-values

For each candidate subnetwork $N_{g}$ of size *m*, the method aims to evaluate whether its genes are enriched for signals of differential expression. To this end, an *unnormalized enrichment score ES_g_* is computed on the basis of the sorted sequence $p_{1}\leq \ldots \leq p_{m}$ of the input *P*-values assigned to the genes in the candidate subnetwork $p_{g},g\in N_{g}$ (subnetwork *P*-values). To compute *ES_g_*, for each position k=1,…,m in the sorted subnetwork *P*-value list, we first calculate the probability ${\tilde{p}}_{g}^{\left(k\right)}$ that, under the null hypothesis of i.i.d. uniform distribution of the input *P*-values, at least *k* of the *p_i_* are lower or equal to *p_k_* using the cumulative distribution function of the binomial distribution:

(1) $${\tilde{p}}_{g}^{\left(k\right)}=\sum_{i=k}^{m}\left(\begin{array}{c} m\\ i \end{array}\right){\left(p_{k}\right)}^{i}{\left(1-p_{k}\right)}^{m-i}.$$

We designate the position in the ordered subnetwork *P*-value list of $N_{g}$ at which minimum ${\tilde{p}}_{g}^{\left(k\right)}$ is achieved by $k^{*}={\text{argmin}}_{k}{\tilde{p}}_{g}^{\left(k\right)}$. The *unnormalized enrichment score ES_g_* is then defined as:

(2) $$ES_{g}=-{\text{log}}_{10}({\tilde{p}}_{g}^{\left(k^{*}\right)}).$$

To correct for biases due to subnetwork size and evaluate statistical significance, the *enrichment P-value*$p_{g}^{⋆}$ is computed by comparing *ES_g_* to a background distribution of $ES_{\text{BG}}$ values obtained on random gene sets of the same size as $N_{g}$:

(3) $$p_{g}^{⋆}=\text{prob}(ES_{\text{BG}}\geq ES_{g}).$$

**Fig. 2:**
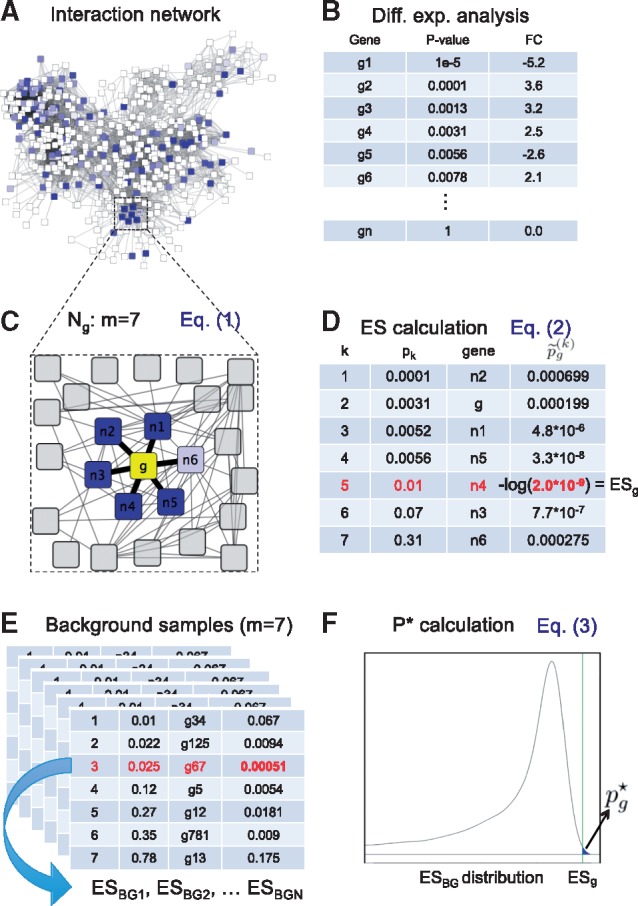
Summary of LEAN. Inputs are (**A**) an interaction network and (**B**) an input *P*-value for each gene in the network, as, e.g. obtained by analysis of differential expression. For any gene *g*, the genes in its direct neighborhood along with their individual input *P*-values are then extracted from the network (**C**). The *P*-values within the neighborhood of *g* are sorted in increasing order and the unnormalized enrichment score *ES_g_* is calculated according to Equation 2 (**D**). To normalize by local subnetwork size, random samples of equal size to $N_{g}$ are drawn from all input *P*-values and a $ES_{\text{BG}}$ value is computed for each of them (**E**). The distribution of $ES_{\text{BG}}$ values is then used to estimate the enrichment *P*-value $p_{g}^{⋆}$, according to Equation 3 (**F**). Used abbreviations: FC = Fold Change (log2) between two conditions

Here, we empirically estimated $p_{g}^{⋆}$ using 10 000 random samples of size *m* from the set of input *P*-values to determine the background distribution of $ES_{\text{BG}}$ values. To correct for the number of local subnetworks being tested, the $p^{⋆}$ values of all candidate subnetworks are subjected to a Benjamini-Hochberg multiple testing correction, yielding the LEAN *P*-values. For each candidate subnetwork with a significant LEAN *P*-value, our implementation of LEAN returns its central gene along with the above mentioned intermediate scores and additional information on the candidate subnetwork. Figure 2 provides an example for the calculation of $p_{g}^{⋆}$ for a candidate subnetwork of size 7.

### 2.4 Interaction network

For use in this study, we employed the murine STRING interaction network (Franceschini *et al.*, 2013). STRING represents one of the largest publicly available collections of protein-protein interaction data (for details on the STRING networks used see supplementary methods). STRING gene interactions originate from different sources of evidence, such as experimental studies of physical interactions, co-expression in public datasets, co-citation in literature and evidence of functional or physical interaction extracted from public databases. Each interaction has an associated global score that reflects the overall strength of evidence for functional interaction. STRING has been used in numerous previous network-based analyses (Abadie *et al.*, 2011; Choudhary *et al.*, 2009; Gaballa *et al.*, 2010; Hofree *et al.*, 2013).

## 3 Results

### 3.1 LEAN detects simulated deregulation of subnetworks

To assess the capacity of the local subnetwork model to detect deregulated pathways, we compared the performance of LEAN to other common pathway analysis methods on simulated data from a statistical model of transcriptomic pathway deregulation. Starting with a high-confidence STRING functional network, we randomly selected a small number of subnetworks as hypothetical pathways, and assigned lower *P*-values to the genes contained in these subnetworks (see supplementary methods for details). We then evaluated the capacity of different methods to recover these simulated pathways. We verified that the graph radius of the simulated subnetworks was substantially larger than 1 (mean subnetwork radius: 2.68  ± 0.7 SD) to ensure that our evaluation dataset is not biased towards overly compact subnetworks, which would confer an advantage to the local subnetwork model (see Supplementary Figs. S1 and S2 for a more detailed evaluation of how the methods perform dependent on subnetwork compactness).

We evaluated the performance of seven approaches: a gene-by-gene approach not using network information, LEAN, the GSEA (Subramanian *et al.*, 2005) enrichment score applied to our definition of local subnetworks (‘local GSEA’), KeyPathwayMiner (KPM) List *et al.* (2016), the jActiveModules method (Ideker *et al.*, 2002), GiGA (Breitling *et al.*, 2004) and RegMOD (Qiu *et al.*, 2010). Performance was measured using Receiver-Operator-Characteristic (ROC) analysis comparing true positive rates (TPR) and false positive rates (FPR) over all possible detection cutoffs with simulated pathway genes defined as positives and genes not contained in simulated pathways as negatives. We varied the characteristics of the simulated pathways using two parameters: To generate low input *P*-values for genes within pathways, the *P*-values of these genes were redrawn from [0, $p_{\text{scale}}$] with probability $p_{\text{enr}}$ per individual gene. In this model, $p_{\text{scale}}$ governs the difference in significance of a pathway gene in comparison to a background gene and $p_{\text{enr}}$ is the expected proportion of pathway genes exhibiting smaller *P*-values than the background genes.

**Fig. 3:**
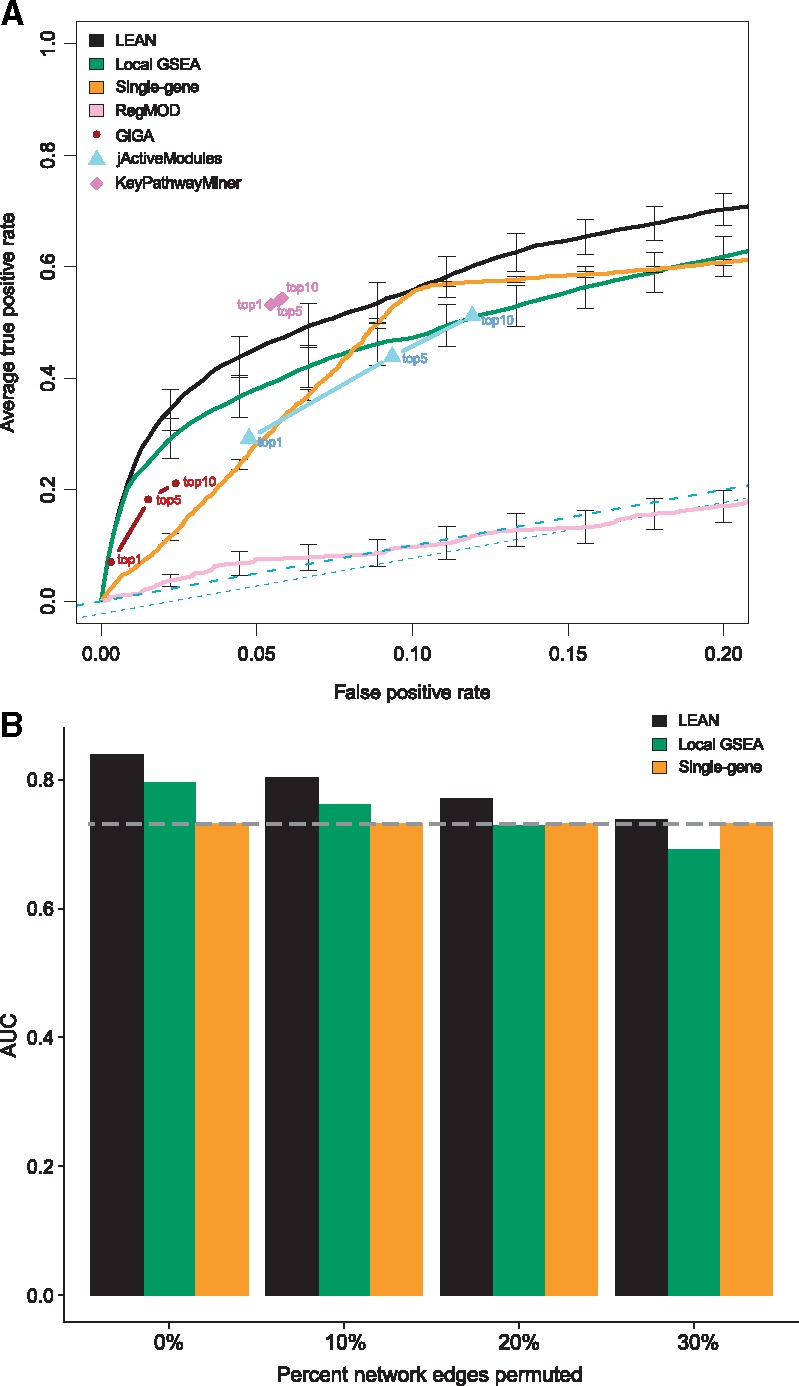
ROC analysis results: Panel A shows average true positive rates (TPR) over 10 separate pathway simulation instances at, given false-positive rates (FPR). Error bars denote standard error of the mean. Average TPR and FPR obtained by 1, 5 and 10 highest-scoring KPM, jActiveModules and GiGA subnetworks, respectively, are shown. All pathway simulations used in the creation of this figure were run with $p_{\text{enr}}=0.5$ and $p_{\text{scale}}=0.1$. Panel B shows areas under the curve (AUCs) after randomly rewiring up to 30% of the network edges

Figure 3A shows ROC curves obtained on pathways with medium *P*-value scaling and medium proportion of significant pathway genes ($p_{\text{enr}}=0.5$; $p_{\text{scale}}=0.1$). Since the KeyPathwayMiner, jActiveModules and GiGA methods do not score individual genes, but entire subnetworks, we could not evaluate them in the same way. For these two methods, we thus obtained the ten highest-scoring subnetworks and computed the TPR and FPR obtained by selecting all genes contained in the highest scoring subnetwork, the five or the ten best scoring subnetworks, respectively. On these data, LEAN *P*-values provide significantly better separation between pathway genes and background genes than most other tested methods (p≤0.05 for comparisons between LEAN and any other ROC evaluation permitting method, DeLong’s method, see supplementary methods for details). This finding was confirmed in an alternative subnetwork model (see Supplementary Fig. S3). KPM was the only method able to provide a slightly better separation than LEAN, possibly aided by the fact that it was run using the optimal choice of *P*-value cutoff (for more details see supplementary methods).

LEAN results were relatively robust to network perturbations in the form of random edge rewiring (see supplementary methods for details) and yielded better performance than single-gene scoring up to a perturbation of 30% of the network edges, which corresponds to a situation where on average only about 58% of the genes in a local subnetwork are kept unchanged (Fig. 3B).

For a more exhaustive evaluation of the impact of pathway simulation parameter values, we computed partial areas under the ROC curve (pAUCs) in the low FPR range (FPR≤0.05) for each of the approaches allowing such evaluation (see Supplementary Fig. S4). We observed that both LEAN and local GSEA substantially outperformed gene-by-gene scoring, especially in cases where the *P*-value improvement of module genes over background genes was subtle. Furthermore, LEAN clearly outperformed the local GSEA score in cases where relatively few of the genes within a pathway were assigned low *P*-values; for high $p_{\text{enr}}$ values the two methods yielded comparable results.

### 3.2 LEAN *P*-values reflect similarity of biological conditions

If statistical profiles resulting from an experiment are reflective of the underlying biology, similar experiments should be expected to lead to similar detections. To examine how much gene- and local subnetwork-level *P*-values conform to this ideal, we evaluated the overlap of genes detected as significant in six publicly available experimental datasets whose biological interpretation we were familiar with.

Four datasets measure the response of cell lines upon stimulation with transforming growth factor beta (TGFβ) and tumor necrosis factor alpha (TNFα) in comparison to appropriate controls. The remaining two datasets respectively compare basal gene expression between two tissue types and a common reference tissue, murine cerebellum. The characteristics of the selected datasets are summarized in Supplementary Table S3. We note that the datasets might contain uncontrolled biases due to variability in cell types and microarray platforms.

We carried out *limma* gene-by-gene analysis of differential expression, as well as LEAN (for details see supplementary methods). To ensure comparable sensitivity, the same cutoff of 0.05 was applied on Benjamini Hochberg-corrected *limma P*-values and equally corrected LEAN *P*-values to obtain 6 lists of significant genes or local subnetwork centers in each case.

**Fig. 4:**
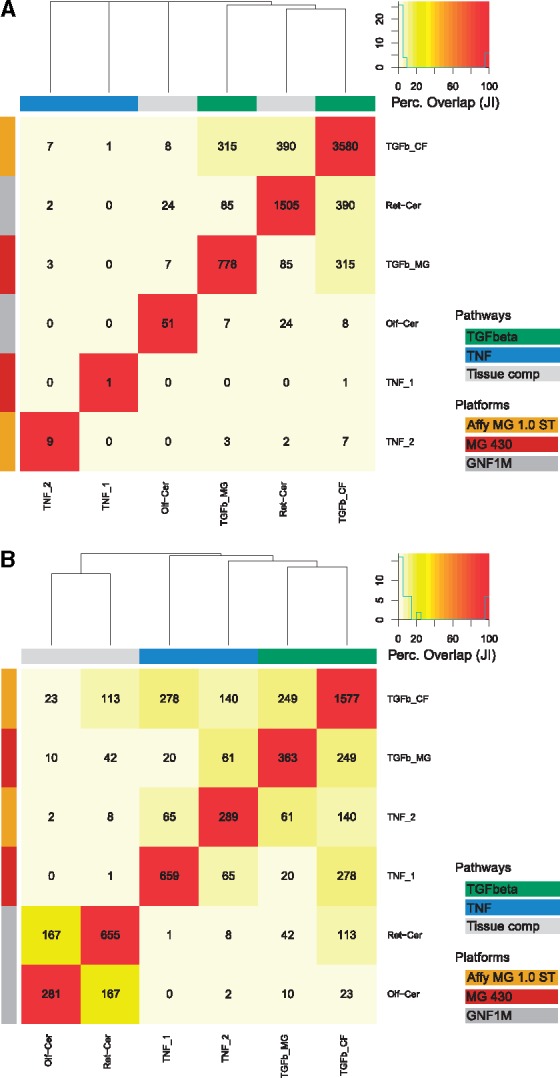
Overlap of significant gene/local subnetwork center lists detected on publicly available datasets: (**A**) Limma gene-by-gene analysis, (**B**) LEAN. Numbers inside cells reflect absolute overlap, color corresponds to Jaccard index (JI). Information about the perturbed pathway and used platform technology are shown as color strips on the top and right of the heat map, respectively. Hierarchical complete linkage clustering of the datasets based on Euclidean distance of Jaccard index profiles is represented as a dendrogram

Figure 4 shows heat maps showing the number of overlapping genes among each pair of lists (see supplementary methods for details). Clustering based on their degree of overlap using limma *P*-values did not yield groups of biologically similar datasets (Fig. 4A), possibly in part due to vastly different number of genes detected as significant in the individual datasets. However, LEAN *P*-values separated stimulation from tissue comparison datasets and—within the stimulation experiments—separated the TGFβ pathway and TNFα pathway related datasets into two groups, irrespective of array platform design used in individual datasets (Fig. 4B).

Enrichment analysis using Enrichr (Chen *et al.*, 2013) of the significant local subnetwork centers detected in both TNFα stimulation datasets returned GO terms and pathways relevant to the studied stimulus: The 65 proteins contained in the overlap showed enrichment of GO biological processes ‘regulation of I-κB kinase/NF-κB signaling’ (*P*-value $4*10^{-40}$, GO:0043122) as well as ‘activation of innate immune response’ (*P*-value $1*10^{-33}$, GO:0002218), congruent with the known role of TNFα in the induction of inflammatory responses via the NFκB signaling cascade (Pober, 2002). Enrichment analysis for the TGF-β and tissue comparison datasets also yielded results congruent with the underlying biology (see supplementary methods and supplemental files 1 and 2).

### 3.3 LEAN unveils striking cellular changes in cerebral cavernous malformations

We applied LEAN to Cerebral Cavernous Malformations (CCM), a condition characterized by vascular malformations of the central nervous system that lead to cerebral hemorrhages. Familial CCM (about 20% of CCM patients) occurs as a condition with autosomal dominant transmission mode caused by loss-of-function mutations in one of the 3 CCM genes: CCM1/KRIT1, CCM2/Malcavernin/OSM and CCM3/PDCD10 (Bergametti *et al.*, 2005; Denier *et al.*, 2004; Laberge *et al.*, 1999; Liquori *et al.*, 2003).

The CCM proteins show no sequence homology and are scaffold proteins without catalytic activity. They have been shown to interact in a ternary complex using CCM2 as a hub. A number of studies have provided insights into CCM protein functions, including cytoskeletal remodelling, cell–cell junction homeostasis, lumen formation and polarization (for a review see (Faurobert and Albiges-Rizo, 2010)). Previously, three inducible, endothelial-specific CCM mouse models (iCCM1-3) (Boulday *et al.*, 2011) have been developed. Inactivation of any of the CCM genes in these mouse models results in the development of vascular lesions strikingly mimicking human CCM lesions. In these mice, as well as in CCM patients, lesions develop in the venous but not in the arterial beds.

To investigate the mechanisms underlying the development of CCM lesions, we performed microarray analyses (for details see supplementary methods) to characterize and compare the transcriptomic profiles of venous tissue of iCCM1-3 and control mice. As the CCM proteins are known to be scaffolding proteins without catalytic activity, the CCM-inactivation phenotype can be assumed to be mediated by faulty or abolished protein-protein interactions and thus presents an interesting case for LEAN.

To this end, gene-by-gene analysis of differential expression between CCM-invalidated and control mice was carried out and resulting non-multiple-testing adjusted *P*-values were subjected to LEAN using the high interaction confidence STRING network (interaction confidence score ≥0.9). This resulted in 68, 211 and 143 local subnetworks significantly enriched for deregulation (LEAN *P*-value ≤0.05) in the comparison of veins invalidated for CCM1, CCM2 and CCM3 versus control tissue, respectively (corresponding lists of local subnetwork centers are supplied in file Supplement3). Since features of the disease are similar independently of the CCM gene ablated (in patients as well as in the mouse models), we examined the subnetworks common to the three groups to determine shared targets and signaling pathways.

Two candidate subnetworks, centered on Coagulation-factor VIII (FVIII) and Von Willebrand Factor (VWF), respectively, were the only ones detected in all three of the CCM invalidation experiments. Applying the approach on a medium-confidence version of the STRING database (interaction confidence score ≥0.4) failed to identify FVIII, but confirmed significance of the VWF candidate subnetwork. Among the other tested subnetwork methods, only the jActiveModules greedy search strategy led to a result containing VWF, but only if a suitable setting of five or more top-scoring networks were used, which results in a large set of at least 82 unprioritized genes (see Supplementary methods and Supplementary Table S2 for details).

**Fig. 5:**
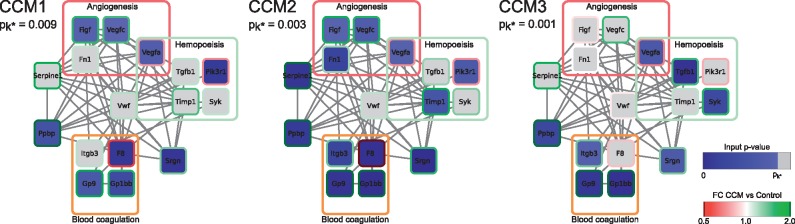
Local subnetwork centered on VWF: The first $k^{*}$ genes contributing most to the significance of the VWF local subnetwork in the three CCM invalidation mouse models are shown as gene networks. Genes are represented as nodes with node border color indicating differential expression. Edges represent functional similarity between pairs of genes with a STRING interaction confidence score ≥0.9. Interactions with VWF have been omitted for better visibility. Annotation of proteins with the three GO biological process terms ‘angiogenesis’, ‘blood coagulation’ and ‘hemopoiesis’ are represented by correspondingly labeled colored frames

Both FVIII and VWF are mostly known for their role in hemostasis. Given the above results and the fact that both the biological functions and the local subnetworks of VWF and FVIII overlap significantly, we focused our further evaluations on VWF. Figure 5 displays the most significantly deregulated genes in the local subnetwork around VWF (genes showing an input *P*-value $\leq p_{k^{*}}$ in at least one of the three CCM mouse models). They are mainly composed of genes involved in angiogenesis, blood coagulation and hemopoiesis pathways (GO biological process terms: GO:0001525, GO:0007596 and GO:0030097 respectively).

VWF itself is a well-established endothelium-expressed gene (for a review see (Lenting *et al.*, 2015)) and involvement of VWF in modulating angiogenesis has been recently proposed (Starke *et al.*, 2011). Using a classical gene-by-gene analysis of our transcriptomic data, ablation of neither one of the three CCM genes lead to VWF mRNA differential expression. GSEA analysis applied to the CCM data did not find any significant gene set in all three CCM invalidations using the MSigDB GO gene set database or the MSigDB curated gene set database (data not shown). Note also that the above mentioned three main biological processes present in the neighborhood of VWF did not achieve significant enrichment under any of the CCM invalidations according to GSEA. A targeted single enrichment test using a single manually compiled list of 56 known VWF-related genes, however, detected significant deregulation of VWF-related genes (hypergeometric test *P*-value $=5*10^{-5}$; for the list of 56 genes see file Supplement 4).

**Fig. 6:**
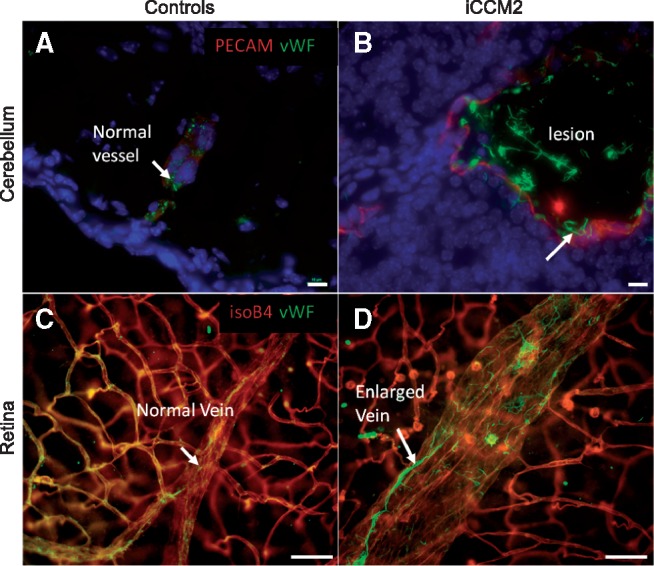
VWF-specific fluorescent staining of mouse tissues shows a dysfunction of the VWF pathway in vivo in the CCM mouse model: The first row shows cerebral sections, the second row whole mount retinas; The first column shows control tissues, the second column tissues from CCM2-ablated animals. Vessels are shown in red (PECAM/isoB4-staining), VWF in green and nuclei in blue. Scale bars: 10 µm (**A**,**B**), 50 µm (**C**,**D**)

These results pointed towards a potential post-transcriptional modification of VWF induced by loss of CCM. Since VWF is synthesized mainly by endothelial cells, we checked for the endothelial VWF protein localization using a VWF-specific fluorescent staining on mouse tissues. In normal vessels of the brain and the retina, we detected small dots of VWF, consistent with normal localization of stored VWF within Weibel-Palade bodies in endothelial cells. Strikingly, at the endothelial surface of cerebral and retinal CCM lesions in CCM2-ablated animals, the dotted staining was replaced by abundant long filaments, so-called VWF strings (Fig. 6). Normal vessels in CCM2-ablated animals showed comparable VWF localization than in control tissues. Altogether, our results clearly confirmed a dysfunction of the VWF pathway *in vivo* in the CCM mouse model.

## 4 Discussion

Despite continuous advances in omics data analysis methods, the interpretation of genome-wide measurements is still difficult in the face of a missing framework of knowledge in which the observed changes can be interpreted. As noted previously, single-gene level interpretation of transcriptomic datasets has a number of major limitations (Subramanian *et al.*, 2005): Reduced sensitivity due to multiple testing, lacking power to detect slight, but concerted changes within molecular pathways, and poor reproducibility of biological results. To overcome these limitations, methods such as GSEA focus on predefined biological pathways instead of single genes, which is thought to increase detection sensitivity and allow easier interpretation of results. Limiting the analysis to predefined pathways, however, precludes detection of novel, previously unknown functions.

Network-based models combine increased sensitivity with the possibility to discover novel functions. The interpretation of subnetworks returned by current network-based models, however, poses a number of challenges: Firstly, validating the involvement of a detected subnetwork in the mechanisms underlying a studied disease is rarely straightforward. Experimental validation typically requires experimental observation or yet better activation/suppression of its activity. Such validation is feasible for single genes and thus also for mechanistically well-understood biological pathways by using a known upstream gene as a proxy. For predicted subnetworks, however, selecting a proxy gene is not straightforward, given the large size of many such predictions (e.g. the 82 or more genes returned by jActiveModules on the CCM data). Secondly, the interpretation of predicted subnetworks is complicated by the fact that most subnetwork methods do not indicate statistical significance of a detection. Moreover, used subnetwork scores are oftentimes not comparable between different experiments. This additional degree of uncertainty reduces the practical utility of such methods. Thirdly, many approaches have limited practical value due to additionally required input data, non-obvious choices of input parameters, dependency on commercial software libraries and/or impracticably long running times (Supplementary Table S2 gives an overview).

### 4.1 The LEAN approach

As other subnetwork-based models, and in contrast to classical gene set enrichment analysis, LEAN combines the ability to detect activity of previously defined pathways with the potential to find active local subnetworks representing biological functions not contained in pathway databases. Activity of known pathways can be detected by LEAN since functional interaction networks—especially when restricted to high-confidence interactions—represent similar knowledge as pathway databases, albeit in a different form. In contrast to other subnetwork-based models, however, the simple LEAN subnetwork model offers the center gene as an evident starting point for further exploration of statistical subnetwork dysregulation. Importantly for practical applications, the simplified local subnetwork model allows the computation of statistically valid *P*-values for all possible local subnetworks in a short time.

The application of LEAN to different public datasets and to transcriptome changes measured in the context of the genetic disease CCM indicate that, despite the simplification of the local subnetwork model, LEAN is capable of detecting previously described pathways within the functional interaction network. One reason for this may be that STRING represents a previously described pathway by adding evidence of functional interaction to all possible pairs of proteins contained therein.

The diversity of evidence represented in functional interaction databases poses a challenge for any subnetwork method. With rapidly advancing technology, the number of known functional interactions across multiple contexts increases, making it unlikely that all known functional interactions of any given protein are involved in the interpretation of any given set of experimental data. This, together with experimental false-positives, can statistically be interpreted as noise that can be expected to dilute the statistical significance of the few relevant interactors. Just as GSEA, LEAN guards against this problem through a statistic that emphasizes the role of the most significant neighborhood *P*-values. The use of exact binomial (instead of Kolmogorov-Smirnoff) statistics ensures sensitivity even in the case of very few significant *P*-values across a wide range of parameters.

A further interesting property of LEAN is its increased detection sensitivity for genes that participate in multiple dysregulated pathways, since signals from all such pathways will contribute to the LEAN *P*-value of the corresponding local subnetworks. A good example of this behavior is VWF, whose local subnetwork covers genes involved in angiogenesis, hemopoiesis and blood coagulation. When analyzed as a set of known pathways, the transcriptomic signals from these pathways each fail individually to meet statistical significance, but the combination of their signal in LEAN makes the joint signal detectable under all three CCM invalidations.

### 4.2 Dependence on pathway compactness

The local subnetwork model is clearly a very restricted representation of biological pathways. This fact, however, does not appear to restrict LEAN’s capability to detect important biological signals. LEAN outperformed a reference method using the common unconstrained subnetwork model on simulated pathways which had a significantly larger average graph radius (2.68, with SEM of 0.078) than the radius of 1 of the local subnetwork model. As expected, we detected a negative correlation between graph radius and LEAN performance in a more detailed subnetwork simulation study (see Supplementary Fig. S1). LEAN did, however, still perform well (average AUC of 0.8) on simulated subnetworks with a radius of 4. The results obtained on publicly available datasets equally indicate that LEAN is capable of detecting biologically plausible pathways and we have no reasons to assume that these pathways represent subnetworks with exceedingly small network radii.

### 4.3 LEAN results on CCM data

LEAN analysis, applied to CCM transcriptomic data, detected two consistently deregulated local subnetworks in all three CCM groups. One of these local subnetworks was centered on VWF, which was not shown to be deregulated itself at the transcription level using gene-by-gene analysis. The hypothesis of a higher-level dysfunction of the subnetwork around VWF was validated *in vivo* in our CCM mouse model. Indeed, while a punctate VWF staining was detected in cerebral and retinal vessels from control animals as well as in normal vessels of CCM2-ablated animals, an accumulation of ultra-large VWF (UL-VWF) multimers was observed on the surface of iCCM2 lesions. Observation of such ultra-large vWF multimers *in vivo* is very unusual since they are proteolyzed very rapidly in normal conditions.

The biological question raised by our study is how this VWF dysfunction relates to the CCM pathogenesis. Our first hypothesis is that VWF strings observed at the surface of the CCM lesions are a consequence of an endothelial injury occurring early on during the lesional process. Indeed, it is important to note that CCM transcriptomic analysis, used as inputs for LEAN, was performed on vessels prior to CCM lesion development.

Another possibility is that the observed VWF strings play a functional role in pathogenesis. UL-VWF multimers were reported *in vivo* under several pathological conditions. VWF strings were detected in human malignant melanoma tissues anchored at the microvessel surface of the tumor, promoting cancer progression (Bauer *et al.*, 2015). Accumulation of UL-VWF multimers has also been reported in patients affected by a thrombotic disorder called Thrombotic Thombocytopenic Purpura (TTP). In the context of CCM, VWF strings could thus promote the formation of thrombi sometimes observed in human CCM caverns.

In the past few years, apart for its role in hemostasis, VWF has been implicated in regulating angiogenesis, smooth muscle cell proliferation and blood-brain-barrier (BBB) properties (Suidan *et al.*, 2013) (for a review, see (Luo *et al.*, 2012)). VWF -/- mice showed a reduction of BBB permeability, associated with an up-regulation of Claudin-5 expression, a major component of the endothelial tight junctions. In human CCM lesions, defective endothelial tight junctions have been reported. We also showed in iCCM2 mouse models that tight junction proteins Claudin-5 and ZO.1 were strongly reduced specifically in the endothelium lining the lesions. Whether abundant VWF strings attached to the lesion surface are involved in the endothelial junction dismantling is so far unknown.

Regardless of the fact that the role of VWF pathway dysregulation remains to be clarified, the present study, by the use of the LEAN method, pointed to an entirely unexpected VWF dysfunction that was confirmed to be relevant for the CCM disease.

### 4.4 Applicability to other types of genomic data

Here, we studied the application of LEAN to transcriptomic data, but we believe that LEAN could equally well be applied to genomic measurements of other biological states, as we see no reason to believe that the local subnetwork model represents an important facet of pathway activity exclusively on the transcriptomic level. Interesting application scenarios would include levels of genetic variants (identification of mutational ‘network hotspots’), proteomics (‘hotspots of proteomic activity’) or integrated datasets comprising multiple data sources. We note that LEAN also applies to incomplete data, as typical in the case of shotgun proteomics: Network nodes without information can just be ignored in the computation of the LEAN statistics.

## 5 Conclusions

We introduced here LEAN, an efficient, exact, exhaustive and parameter-free method that complements single-gene analysis and circumvents common problems in gene set enrichment and network-based analysis through a restricted local subnetwork model. In our evaluation on simulated data, LEAN–despite its restricted subnetwork model–performs better than most other tested methods. Applied to the transcriptome of murine models for the genetic disease CCM, LEAN predicted the involvement of VWF, which had neither been implicated in the pathophysiology of the disease, nor shown detectable deregulation on the mRNA level. Experimental evaluation of VWF protein in vivo confirmed abnormal VWF conformation and localization in the mouse model of CCM disease.

## Supplementary Material

Supplementary Data
