# Supplementary material for: Network-based analysis of omics data: the LEAN method
Source: Bioinformatics. 2016 Dec 6;33(5):701–9. doi: 10.1093/bioinformatics/btw676 (PMC5408824; doi:10.1093/bioinformatics/btw676)
Supplement: Supplementary Data [file btw676_supp.zip › Supplement_R2.pdf]

BIOINFORMATICS
Vol. 00 no. 00 2016
Pages 1–6

Supplementary material for ”Network-based analysis of omics data: The LEAN method”

Frederik Gwinner [1,2,*], Gwénola Boulday [1,2,*], Claire Vandiedonck [3,4], Minh Arnould [1,2], Cécile Cardoso [1,2], Iryna Nikolayeva [5,6,7], Oriol Guitart-Pla [5], Cécile V. Denis [8], Olivier D. Christophe [8], Johann Beghain [6,9], Elisabeth Tournier-Lasserve [1,2,10,†], Benno Schwikowski [5,†]

[1] Univ Paris Diderot, Sorbonne Paris Cité, UMRS 1161, F-75010 Paris, France
[2] INSERM, U1161, F-75010 Paris, France
[3] Univ Paris Diderot, Sorbonne Paris Cité, UMRS 958, F-75010 Paris, France
[4] INSERM, U958, F-75010 Paris, France
[5] Systems Biology Lab, C3BI, USR 3756 Institut Pasteur/CNRS, Institut Pasteur, F-75015 Paris, France
[6] Functional Genetics of Infectious Diseases Unit, Institut Pasteur, F-75015 Paris, France
[7] Univ Paris-Descartes, Sorbonne Paris Cité, F-75006 Paris, France
[8] Unité 1176, INSERM, Univ Paris-Sud, Université Paris-Saclay, F-94270 Le Kremlin-Bicêtre
[9] Genetics and Genomics of Insect Vectors, Institut Pasteur, F-75015 Paris, France
[10] AP-HP, Groupe Hospitalier Saint-Louis Lariboisière-Fernand-Widal, F-75010 Paris, France

Received on XX/XX/XXXX

Associate Editor: XXXXXXX

SUPPLEMENTARY METHODS

STRING data extraction

A full list of interactions between murine proteins was downloaded from the STRING website (http://string-db.org, database version 9.1). Two versions of the network were created by filtering for interactions with high confidence (STRING combined confidence score ≥ 0.9) or medium confidence (STRING combined confidence score ≥ 0.4). Ensembl protein ids supplied by STRING were mapped to Ensembl gene ids using the Ensembl biomart tool (version 67). In total, 7,279 and 17,458 of the 22,313 genes present on the Affymetrix Mouse Gene 1.0 ST microarray could be mapped to a protein present in the high and medium-confidence interaction network and the resulting networks contained 63,617 and 386,749 interactions between those proteins, respectively.

Simulation of deregulated subnetworks

The subnetwork simulation study was based on the high-confidence version of STRING described above.

Generation of subnetworks Each subnetwork was generated by first drawing its size uniformly at random between 10 and 50. A single gene was selected with uniform probability as a seed subnetwork. This seed subnetwork was extended to the desired size by iteratively picking one gene in the subnetwork, with a probability proportional to its degree in the complete network, and adding one of its neighbors – randomly selected – to the subnetwork. This process was repeated 10 times to generate 10 (possibly overlapping) subnetworks.

Generation of p-values We generated p-values according to a statistical model of transcriptomic deregulation. Genes outside of the simulated subnetworks were assigned a uniform random p-value in [0, 1]. Genes within the subnetworks were, with probability p_{enr}, reassigned a p-value drawn from $[0, p_{\text{scale}}]$. p_{scale} and p_{enr} are thus the two input parameters determining the p-value distribution of simulated pathway genes in our pathway simulation model.

Evaluation To characterize the performance of LEAN, we carried out ROC analysis on the results of LEAN applied on the simulated p-values as described earlier. As the GSEA method (Subramanian *et al.*, 2005) has previously been used to evaluate the influence of transcription factors on differential expression (e.g. in (Wang *et al.*, 2009)) we decided to include the GSEA enrichment score (”local GSEA”) computed on the same local subnetwork as used by LEAN. RegMOD does not return individual subnetworks but rather an ”active network score” per gene, which we used in the ROC analysis. The average TPR curves for LEAN, local GSEA and RegMOD, shown in Figure 3 of the main manuscript have been generated by running 10 separate subnetwork simulation instances, each one containing 10 individual simulated subnetworks. As the KeyPathwayMiner, jActiveModules and GiGA methods return individual subnetworks applying a standard ROC analysis to these results was not possible. Instead we extracted, for each of the

*these authors contributed equally to this work
†to whom correspondence should be addressed

© Oxford University Press 2016.

ten simulation instances, the ten highest-scoring subnetworks and computed TPRs and FPRs obtained by considering genes contained in either only the highest-scoring subnetwork, the five best-scoring subnetworks or the ten highest-scoring subnetworks as predicted. Figure 3 shows the corresponding average TPR and FPR obtained over the ten simulation instances.

Statistical significance of differences in the ROC analysis DeLong's method as implemented in the function `roc.test` (one-sided test, with default parameters) of the R package pROC (Robin *et al.*, 2011) was used to assess statistical significance of differences between AUCs obtained using approaches that allow a full ROC analysis (LEAN, local GSEA, single-gene scoring and RegMOD). When applied to each individual simulation instance separately, the tests confirmed a significant increase of LEAN performance over the local GSEA and RegMOD approach in all 10 simulation instances ($p < 0.006$ for each of the 10 instances). In the comparison to the single-gene scoring approach, 8 out of 10 instances showed a significant improvement of LEAN ($p < 0.00065$) and two instances narrowly missed significance ($p < 0.07$). When pooling the 10 simulation instances by concatenating the gene scores of individual instances, all comparisons achieved significance ($p < 2.2 * 10^{-16}$).

Simulation study without preferential attachment To assess the impact of the subnetwork model on method performance, we repeated our simulation study with an alternative subnetwork model. To this end, the step "Generation of subnetworks" described above was modified such that new nodes were selected randomly with uniform probability. The remaining steps of the simulation study were carried out without changes. Figures S3 and S2 show the results of this simulation study (cf. Figure 3 in the main text and Figure S1, respectively).

Robustness to network perturbations To assess robustness of our results to network perturbations, we started from the p-values generated in the subnetwork simulation study with preferential attachment described above. The STRING network was perturbed by randomly rewiring a fixed percentage of its edges (0% to 30%) using the function `rewire` from the igraph R package. LEAN as well as the local GSEA score were then computed by calling the respective functions with the generated subnetwork and background p-values and the perturbed versions of the STRING network. On these data, rewiring 30% of network edges led to a substantial perturbation of the local subnetworks. On average, each local subnetwork retained only about 58% of its original genes. To put this into perspective: In the case of the data presented in Figure 3, where the parameter p_{enr} was set to 0.5, the task given to LEAN can be interpreted as finding modules, of which, statistically, only half of the genes are expected to have a measurable input p-value difference to background genes, and almost 30% were not actual neighbors in the unperturbed network. As the network information is not used by the single-gene approach, it is not affected by the network perturbations, but is still slightly outperformed by LEAN.

Analysis of publicly available data sets

Transcriptome analysis Pre-normalized expression data were retrieved from GEO using the R Bioconductor package *GEOquery* (Davis and Meltzer, 2007). Replicate samples for the two conditions to be compared (as detailed in Table S3) were extracted and subjected to a differential expression analysis using the empirical Bayes method parameterized by group-means implemented in the *limma* package. Probeset identifiers were mapped to Ensembl gene identifiers using the respective manufacturer-supplied annotation if available and by using the Ensembl biomart mapping tool otherwise. In case of multiple probesets matching a single gene identifier, the probeset with maximal average expression over all samples was used. Two versions of the limma results were created, one in which only genes contained in the STRING network were used in the p-value calculation (results shown in Figure 4A of the main manuscript) and one in which the analysis was based on all genes regardless of their presence in STRING. The second analysis produced comparable p-values and did not lead to a better grouping of biologically similar data sets than the first (Data not shown). For both of the gene-by-gene analyses, genes with a Benjamini-Hochberg adjusted p-value ≤ 0.05 were treated as statistically significant without taking the measured mean fold changes into account.

LEAN For the application of LEAN to public data sets, gene-by-gene p-values of individual genes were mapped to proteins in the medium-stringency version (combined confidence score ≤ 0.4) of the STRING network as described above. LEAN was applied separately for each data set and central proteins of local subnetworks with LEAN p-value or limma p-value ≤ 0.05 deemed significant for LEAN and limma analysis, respectively. Pairwise overlap between lists of significant genes obtained on different data sets was measured by the *Jaccard index* ($\frac{|A \cap B|}{|A \cup B|}$) and heat maps depicting these pairwise overlaps generated with the R *gplots* library function *heatmap.2* using a hierarchical complete linkage clustering based on euclidean distances over Jaccard index profiles for dendrogram generation.

Enrichment analysis Enrichment analysis using Enrichr (Chen *et al.*, 2013) of the significant local subnetwork centers detected in both tissue comparisons (both TGFβ stimulation, and both TNFα stimulation data sets, respectively) returned GO terms and pathways relevant to the studied processes:

The 167 proteins significant in both tissue comparison experiments were enriched for cerebellum-specific gene expression (p-value $1 * 10^{-13}$, Mouse Gene Atlas) and "synaptic transmission" (p-value $2 * 10^{-41}$, GO biological process id: GO:0007268).

The overlap of TNFα stimulation data sets contained 65 proteins and showed enrichment of "TNFα NF-κB Signaling Pathway" (p-value $2 * 10^{-20}$, WikiPathways) and GO biological processes "regulation of I-κB kinase/NF-κB signaling" (p-value $4 * 10^{-40}$, GO:0043122) as well as "activation of innate immune response" (p-value $1 * 10^{-33}$, GO:0002218), congruent with the known role of TNFα in the induction of inflammatory responses via the NFκB signaling cascade (Pober, 2002).

The 249 proteins significant in the two TGFβ stimulation data sets showed enrichment for "TGF Beta Signaling Pathway" (p-value $9 * 10^{-16}$, WikiPathways), the GO biological process terms "response to transforming growth factor beta" (p-value $1 * 10^{-20}$, GO:0071559), "tissue morphogenesis" (p-value $5 * 10^{-23}$, GO:0048729) and "angiogenesis" (p-value $2*10^{-28}$, GO:0001525), consistent with the well-known role of the TGFβ pathway in

both normal tissue development (Wu and Hill, 2009) and cancer progression (Derynck *et al.*, 2001). Please refer to file Supplement1 for the proteins contained in the respective overlaps of significant local subnetwork centers and to file Supplement2 for the full results of the enrichment analysis.

CCM invalidation experiments

Sample hybridization and intensity read-out Sample hybridization and intensity read-outs were performed at the "IGBMC Microarray and Sequencing platform" in Strasbourg (for details see Supplementary Material). Data normalization and statistical analysis was carried out in R. Data were normalized using the *rma* function from the *affy* package and the appropriate Ensembl gene library CDF file (version 16.0.0) from Brainarray (Dai *et al.*, 2005). Quality controls included the visualization of standard deviation versus mean expression, density distribution of normalized expression values as well as the computation of Pearson correlations between arrays. All of these controls showed behaviour indicative of high-quality samples and hybridizations. Differential gene expression versus control conditions were determined using the empirical Bayes method parameterized by group-means implemented in the *limma* package (Smyth, 2004).

LEAN For the application of LEAN to CCM invalidation data, limma gene-by-gene p-values of individual genes were mapped to proteins in the high-stringency version of the STRING network as described above. LEAN was applied separately for each CCM invalidation and central proteins of local subnetworks with LEAN p-value ≤ 0.05 deemed significant.

Comparison to other network-based approaches

As mentioned in the main text, many previously published network-based approaches are not readily applicable to any network and data set for various reasons. Table S2 gives an overview of the applicability and run times of the tested approaches. In the following, we will give details on the required transformations of the input data and input parameter values for the individual approaches:

- **BioNet** The BioNet R-package (v1.29.1) was downloaded from Bioconductor. We ran BioNet using the heuristic scoring function as the exact solution requires a CPLEX license. The first step of applying BioNet consists in fitting a beta-uniform mixture model (bum model) to the input p-values to transform them into a score used subsequently in the Prize-collecting Steiner tree (PCST) problem solved by BioNet (these scores are used as node prizes in the PCST; unusually low p-values are represented with positive values). As the input p-values generated in our model simulation study do not follow a bum model, unsurprisingly the bum model fitting failed on all instances of the simulation study and thus did not allow us to apply BioNet in this context. On the CCM data, the bum model fitting succeeded for the CCM2 and CCM3 input p-values, but failed in the case of the CCM1 data. On the CCM2 and CCM3 data, BioNet found subnetworks of size 88 and 108, respectively. Neither of the two contained VWF. The overlap between the two detected subnetworks consisted of 21 genes 6 of which were direct neighbors of VWF in the STRING network.
- **DEGAS** We downloaded the java executable of the MATISSE tool suite (v1.1) from the authors website. As DEGAS requires expression data as input, we were unable to apply it in the context of the module simulation study. The DEGAS tool in a first step requires setting a p-value cutoff for a binary classification into deregulated/non-deregulated genes. We tested the default cutoff of 0.05 as well as a relaxed cutoff of 0.1 on the CCM2 data. The remaining parameters to be set are the minimum size of subnetworks to consider k and the number of allowed samples which are allowed to have less than k deregulated genes l. For application to the CCM2 data, we tested values for $k \in \{10, 20, 50, 100, 200, 300, 400, 500\}$ and $l \in \{0, 1\}$, as in our example there are only three case samples. For $l = 0$ no significant modules with more than a single gene were found. For $l = 1$, none of the significant subnetworks contained VWF (the largest significant subnetwork contained 126 genes).
- **GiGA** The perl executable supplied by the authors was downloaded from the journal website. For the module simulation study, we set the maximum subnetwork size to 50 (as the modules were simulated to contain at most 50 genes each) and extracted the ten highest-scoring subnetworks. In the case of the CCM data, we used the default maximum subnetwork size of 20 and again extracted all significant subnetworks ($p < 0.0001$) as suggested by the GiGA implementation. None of the detected significant subnetworks on the three CCM invalidations contained VWF, although the highest-scoring detected subnetwork in the case of the CCM2 invalidation was composed of 17 genes, 8 of which were direct neighbors of VWF in the STRING network.
- **GXNA** The GXNA (v2.0) source code was downloaded from the downloads website, compiled, and executed with the options `-algotype 1` to search for subnetworks, `-flexSize` for variable size, and `-nPerms 10000` to ensure sufficient accuracy of empirical p-values. These parameters resulted in no detection of a significant subnetwork (the smallest resulting adjusted p-value was 0.101).
- **hotnet2** The hotnet2 (v1.0.1) source code was downloaded from the tools website and installed as specified in the installation instructions. In order to apply hotnet2 to a new network, the first step consists in calculating so-called influence matrices, which serve to estimate significance of subnetworks by comparing their scores to scores obtained on permuted versions of the network (the authors supply pre-computed influence matrices for HPRD, IREFand other networks). With default settings (i.e. 1000 network permutations), the calculation of those influence matrices took 30 minutes per permutation. Based on this estimate, it would have taken approximately three weeks ($1000 * 0.5h = 500h \approx 21d = 3w$) to finish these calculations. We thus considered the tool not readily applicable on the STRING network. In principal, a speed up of these calculations by parallelization should be possible, but the supplied python code did not offer an option for parallel execution.

- **ILP/Branch&Cut** As the implementation of the Branch and Cut strategy requires a CPLEX license we were unable to test it on our data.
- **jActiveModules** To ease exploration of input parameters, we used a standalone java version of jActiveModules developed in-house. jActiveModules - on both the module simulation study and the CCM data - was run using either the simulated annealing strategy (default parameters and 250.000 iterations) or the greedy search strategy (default parameters). In both cases the ten highest-scoring subnetworks were extracted. As previously described, the simulated annealing strategy showed a tendency to return overly large subnetworks: in the case of the CCM data the best-scoring subnetwork contained between 1246 and 1588 genes. The greedy search surprisingly yielded much more compact results. When applied to the CCM data, the highest-scoring subnetworks contained 298, 215 and 219 genes, respectively for the CCM1, CCM2 and CCM3 data sets. The overlap of these three subnetworks contained 21 genes, including 5 of the 56 direct neighbors of VWF, but not VWF itself. Considering any of the genes contained in the five highest-scoring subnetworks per CCM experiment led to prediction of 551, 451 and 447 genes in the CCM1, CCM2 and CCM3 data sets, respectively. The overlap of these sets contained 82 genes, including VWF. In detail, VWF was contained in the fifth-best scoring subnetwork of the CCM1 results, the best-scoring subnetwork of the CCM2 results and the second-best scoring subnetwork of the CCM3 results.
- **KeyPathwayMiner (KPM)** For evaluation of KPM on the module simulation data, we used the Web-based version of KPM List *et al.* (2016) with a numerical matrix (of dimension number of genes x 1) composed of the simulated p-values as input. We set the p-value cutoff to the value of p_{scale}, so that genes with a p-value of less than 0.1 were counted as "active". Note that, in practical applications, this (optimal) value of the cutoff parameter is typically not available. Since we only had a single condition, the parameter l was fixed at 0. We tested three evenly-spaced values for the input parameter k ($k = 1, 6, 11$) within the allowed limits (the maximum value for k is set to 11 by the web server). The number of desired pathways was set to 10, representing an optimal (in applications, unknown) choice given the characteristics of the simulated data. We chose the default search strategy, a greedy search with individual node exceptions (INES). The best results in terms of TPR and FPR were obtained using $k = 11$. The corresponding results are represented in Figure 3. KPM generally returned pathways that were largely overlapping with each other (on average 95% of all genes contained in the overlap of the 10 returned pathways were already contained in the best scoring pathway), possibly due to the used search strategy, which nevertheless contained a high proportion of the simulated module genes while producing relatively few false positives.
For application of KPM to the CCM data, we uploaded the three p-value vectors determined using a limma analysis of the CCM expression data as separate data sets. We then ran KPM using $l = 0$, $k = 1, 6, 11$ and p-value cutoffs at $0.05, 0.01$ or 0.001. The three data sets were combined using an "AND" logic. We used the INES greedy search strategy to allow detection of "hub genes" such as VWF. As described in the case of the module simulation study, the returned pathways showed a large overlap. We thus only extracted the union network created by merging the 10 returned pathways for each parameter combination. The resulting networks contained 1533 ± 26 ($p = 0.05$), 537 ± 36 ($p = 0.01$) and 141 ± 36 ($p = 0.001$) genes, with higher values of k leading to slightly bigger networks. None of the resulting networks contained VWF.
- **RegMOD** The Matlab code supplied by the authors was downloaded from the journal website. As RegMod requires "activity scores" (higher scores indicating more significant dysregulation), we transformed the module simulation input p-values into such scores by taking the negative logarithm. For the two input parameters β and C, we tested values similar to the ones used in the RegMOD paper ($\beta \in \{1, 2, 3, 4\}$ and $C \in \{0.5, 1, 1.5\}$). The best performance in the module simulation study was achieved using the parameters $\beta = 3$ and $C = 1$. As the performance of RegMOD on the module simulation study was very disappointing and not competitive with any of the other methods, we did not apply it to the CCM data.
- **PinnacleZ** The PinnacleZ plugin (v1.1) for Cytoscape 2.6 was downloaded from the Cytoscape App Store. As PinnacleZ requires expression data as input, we were unable to apply it to the module simulation study. Run with default parameters on the CCM2 data (in which the VWF subnetwork shows most significant deregulation), PinnacleZ identified 164 significant subnetworks (in total $\tilde{6}50$ genes), none of which contained VWF.

SUPPLEMENTARY MATERIAL

Experimental Material and Methods

Mice The iCCM1-3 mouse lines were reported previously (Boulday *et al.*, 2011; Maddaluno *et al.*, 2013). Tamoxifen induction (20mg/kg) was performed using a single injection at P2. All procedures described in this study were in full accordance with the Institutional Animal Care and Use Committee "Lariboisiere-Villemin" (Committee number 9, Paris, France).

Immunofluorescence Whole-mount retinas and 10 μm thick sections of OCT-embedded mouse brains were stained for immunofluorescence microscopy, as previously described (Boulday *et al.*, 2011). The following antibodies (Abs) were used: rat anti-PECAM (MEC13.3, BD Pharmingen); rabbit anti-VWF (A0082, Dako) ; Alexa fluor594-conjugated anti-rat (Molecular Probe); AlexaFluor488-conjugated donkey anti-rabbit (Molecular Probes). To visualize retinal vessels, retinas were incubated with biotin-conjugated isolectin-B4 (AbCys) followed by Cy^{TM}3-streptavidin (Amersham). A Nikon Eclipse 80*i* microscope was used to analyze immunofluorescence stainings.

Sample hybridization and intensity read-out for the CCM invalidation data Biotinylated single strand cDNA targets were prepared, starting from 200 ng of total RNA, using the Ambion WT Expression Kit and the Affymetrix GeneChip WT Terminal Labeling Kit according to Affymetrix recommendations. Following

fragmentation and end-labeling, 1.9 μg of cDNAs were hybridized for 16 hours at 45°C on GeneChip Mouse Gene 1.0 ST arrays (Affymetrix) interrogating 28,853 genes (designed on UCSC mm8, NCBI build 36) represented by approximately 27 probes spread across the full length of the gene. The chips were washed and stained in the GeneChip Fluidics Station 450 (Affymetrix) and scanned with the GeneChip Scanner 3000 7G (Affymetrix) at a resolution of 0.7 μm. Raw data (.CEL Intensity files) were extracted from the scanned images using the Affymetrix GeneChip Command Console (AGCC) version 3.2.

Key features of LEAN and previous subnetwork analysis approaches

- Theoretical key features of LEAN in comparison to previous subnetwork analysis approaches: Table S1
- Practical considerations on LEAN and previous subnetwork analysis approaches: Table S2

Pathway simulation study

- Dependency of LEAN ROC-performance on network statistics of the simulated subnetworks: Figure S1
- Dependency of LEAN ROC-performance on network statistics of subnetworks simulated without preferential attachment: Figure S2
- ROC-performance on subnetworks simulated without preferential attachment: Figure S3
- ROC analysis results of LEAN and "local GSEA" expressed as percent improvement in terms of pAUC (FPR ≤ 0.05) over gene-by-gene scoring: Figure S4
- Type-I error rate estimate of LEAN applied on randomized input p-value list: Figure S5

Analysis of public data sets

- Overview of publicly available data sets used in the evaluation of LEAN: Table S3
- Lists of proteins contained in the individual data set overlaps: file `Supplement1.xlsx`
- Complete results of the Enrichr enrichment analysis: file `Supplement2.xlsx`

CCM invalidation

- List of significant local subnetworks detected in the CCM invalidation experiments: file `Supplement3.xlsx`
- Manually compiled list of VWF-related genes used for enrichment analysis: file `Supplement4.xlsx`

LEANR code

- R package LEANR implementing the method containing a vignette to allow reproduction of the results on the CCM data (type `vignette('CCM-data')` in R) and a vignette allowing reproduction of the subnetwork simulation study (type `vignette('subnet-sim')` in R): file `LEANR_1.4.7.tar.gz`

REFERENCES

Boulday, G., Rudini, N., Maddaluno, L., Blécon, A., Arnould, M., Gaudric, A., Chapon, F., Adams, R. H., Dejana, E., and Tournier-Lasserve, E. (2011). Developmental timing of ccm2 loss influences cerebral cavernous malformations in mice. *J Exp Med*, **208**(9), 1835–47.

Chen, E., Tan, C., Kou, Y., Duan, Q., and Wang. . . , Z. (2013). Enrichr: interactive and collaborative html5 gene list enrichment analysis tool. *BMC* . . . .

Chodosh, L., Liu, Z., and Pan, T. (2012). Methods and systems for quantitatively assessing biological events using energy-paired scoring. WO Patent App. PCT/US2011/052,329.

Dai, M., Wang, P., Boyd, A. D., Kostov, G., Athey, B., Jones, E. G., Bunney, W. E., Myers, R. M., Speed, T. P., Akil, H., Watson, S. J., and Meng, F. (2005). Evolving gene/transcript definitions significantly alter the interpretation of genechip data. *Nucleic Acids Research*, **33**(20), e175.

Davis, S. and Meltzer, P. S. (2007). Geoquery: a bridge between the gene expression omnibus (geo) and bioconductor. *Bioinformatics*, **23**(14), 1846–7.

Derynck, R., Akhurst, R. J., and Balmain, A. (2001). Tgf-beta signaling in tumor suppression and cancer progression. *Nat. Genet.*, **29**(2), 117–29.

Essen, D. V., Engist, B., Natoli, G., and Saccani, S. (2009). Two modes of transcriptional activation at native promoters by nf-b p65. *Plos Biol*, **7**(3), e73.

Ifkovits, J. L., Addis, R. C., Epstein, J. A., and Gearhart, J. D. (2014). Inhibition of tgf signaling increases direct conversion of fibroblasts to induced cardiomyocytes. *PloS one*, **9**(2), e89678.

List, M., Alcaraz, N., Dissing-Hansen, M., Ditzel, H. J., Mollenhauer, J., and Baumbach, J. (2016). Keypathwayminerweb: online multi-omics network enrichment. *Nucleic Acids Res*, **44**(W1), W98–W104.

Maddaluno, L., Rudini, N., Cuttano, R., Bravi, L., Giampietro, C., Corada, M., Ferrarini, L., Orsenigo, F., Papa, E., Boulday, G., Tournier-Lasserve, E., Chapon, F., Richichi, C., Retta, S. F., Lampugnani, M. G., and Dejana, E. (2013). Endmt contributes to the onset and progression of cerebral cavernous malformations. *Nature*, **498**(7455), 492–6.

Pober, J. S. (2002). Endothelial activation: intracellular signaling pathways. *Arthritis Res*, **4 Suppl 3**, S109–16.

Rajashekhar, G., Grow, M., Willuweit, A., Patterson, C. E., and Clauss, M. (2007). Divergent and convergent effects on gene expression and function in acute versus chronic endothelial activation. *Physiological Genomics*, **31**(1), 104–113.

Robin, X., Turck, N., Hainard, A., Tiberti, N., Lisacek, F., Sanchez, J.-C., and Mller, M. (2011). proc: an open-source package for r and s+ to analyze and compare roc curves. *BMC Bioinformatics*, **12**, 77.

Smyth, G. K. (2004). Linear models and empirical bayes methods for assessing differential expression in microarray experiments. *Statistical Applications in Genetics and Molecular Biology*, **3**(1), 1–28.

Su, A. I., Wiltshire, T., Batalov, S., Lapp, H., Ching, K. A., Block, D., Zhang, J., Soden, R., Hayakawa, M., Kreiman, G., Cooke, M. P., Walker, J. R., and Hogenesch, J. B. (2004). A gene atlas of the mouse and human protein-encoding transcriptomes. *Proc Natl Acad Sci USA*, **101**(16), 6062–7.

Subramanian, A., Tamayo, P., Mootha, V. K., Mukherjee, S., Ebert, B. L., Gillette, M. A., Paulovich, A., Pomeroy, S. L., Golub, T. R., Lander, E. S., and Mesirov, J. P. (2005). Gene set enrichment analysis: a knowledge-based approach for interpreting genome-wide expression profiles. *Proc Natl Acad Sci USA*, **102**(43), 15545–50.

Wang, K., Saito, M., Bisikirska, B. C., Alvarez, M. J., Lim, W. K., Rajbhandari, P., Shen, Q., Nemenman, I., Basso, K., Margolin, A. A., Klein, U., Dalla-Favera, R., and Califano, A. (2009). Genome-wide identification of post-translational modulators of transcription factor activity in human b cells. *Nat Biotechnol*, **27**(9), 829–837.

Wu, M. Y. and Hill, C. S. (2009). Tgf-beta superfamily signaling in embryonic development and homeostasis. *Developmental Cell*, **16**(3), 329–343.

Method	Arbitrary subnetwork model	Assessment of statistical significance	Polynomial-time tractable	Guarantees optimal solution	Parameter-free	Minimum number of samples used in evaluations	Reference
KeyPathwayMiner	•	—	❍	❍	—[1]	70	Alcaraz et al. (2012), Integr. Biol.
DEGAS	•	•	—	—	—[2]	16	Ulitsky et al. (2010), PLoS one
HotNet	•	•	•	—	—[3]	8	Vandin et al. (2012), Pac. Symp. Biocomput.
BioNet	•	—	❍	❍	•[4]	194	Beisser et al. (2010), Bioinformatics
GiGA	•	•	•	—	—[5]	7 conditions	Breitling et al. (2004), BMC Bioinformatics
jActiveModules	•	—	—	—	—[6]	20	Ideker et al. (2002), Bioinformatics
ILP/Branch & Cut	•	•	—	•	—[7]	24	Backes et al. (2012), Nucleic Acids Res.
RegMod	•	—	•	—	—[8]	115	Qiu et al. (2010) BMC Bioinformatics
GXNA	•	•	❍	❍	—[9]	8	Nacu et al. (2007), Bioinformatics
PinnacleZ	•	•	•	—	—[10]	268	Chuang et al. (2007), Molecular Systems Biology
**LEAN**	**—**	**•**	**•**	**•**	**•**	**6**	**This study**

• = yes; — = no; ❍ = conditional

[1] k (allowed gene exceptions) and l (allowed sample exceptions)
[2] k (necessary subnet genes) and l (allowed sample exceptions)
[3] delta (weight cutoff for edges which defines connected components evaluated as solutions); "optimal default value" given
[4] Hyper-parameter, automatically estimated from data
[5] Maximal subnet size
[6] Diverse parameters related to simulated annealing
[7] Fixed subnetwork size
[8] theta (cutoff on node activity after "smoothing")
[9] Multiple choices at multiple steps of the algorithm
[10] d (maximal distance allowed in extension process) and r (subnetwork score improvement rate used as stopping criterion for subnetwork extension process)

Table S1. Theoretical features of LEAN in comparison to previous subnetwork analysis approaches

Method	Implementation	Applicable simulation?	Performance simulation	Applicable CCM data?	VWF found ?	Run time high-confidence STRING network	Remarks
KeyPathwayMiner	Web server	•	**> LEAN**	•	—	10 s - 2 m	
DEGAS	Java	—	—	•	—	2 – 45 m (dependent on k)	Needs expression data
HotNet	Python	❍	❍	❍	❍	❍	Necessary preprocessing step takes ~3 weeks to complete
BioNet	R package	—	—	•	—	~3 m	Beta-uniform mixture model fitting fails on simulation study and CCM1 data
GiGA	Perl	•	> single gene (AUC = 0.73)	•	—	~3.5 m	
jActiveModules	Java	•	≈ single gene (AUC = 0.73)	•	•	SA[1]: ~3 | ~12 m<br>GS[3]: ~1 | 6-7 m	Run times are given as: Standalone | Cytoscape[2]
ILP/Branch & Cut	Binary executable	—	—	❍	❍	❍	Executable needs CPLEX license
RegMod	MatLab	•	AUC = 0.61	•	❍	8-10 m	
GXNA	C++	—	—	•	—	~1 m	Needs expression data
PinnacleZ	Cytoscape	—	—	•	—	~1 m	Needs expression data
**LEAN**	**R package**	**•**	**AUC = 0.82**	**•**	**•**	**~1.5 m (1k BGs)**<br>**~10 m (10k BGs)**	**1000 BG[4] permutations used in simulation study; 10.000 on CCM data**

• = yes; — = no; ❍ = unclear/not tested

[1] SA = Simulated Annealing
[2] We developed a standalone version of jActiveModules with faster run times than the corresponding Cytoscape plugin.
[3] GS = Greedy Search
[4] BG = Background

Table S2. Practical considerations of LEAN in comparison to previous subnetwork analysis approaches: Calculation times were determined on a MacBook Air (late 2012) with an 1.5 GHz Intel Core i5 and 8GB of RAM using up to 3 of the 4 cores in parallel if allowed by the respective tools.

Shorthand	Reference	GEO acc.	Rep.	Array	Cell type	Description
TNF 1	(Rajashekhar *et al.*, 2007)	GSE4518	4	Mouse Genome 430	Endothelial cells (in vitro)	Stimulation with soluble TNFα 20 ng/ml for 4 hours vs control
TNF 2	(Essen *et al.*, 2009)	GSE12697	3	Mouse Gene v1.0 ST	Fibroblasts (in vitro)	Stimulation with soluble TNFα 5 ng/ml for 1 hour vs control
TGFb CF	(Ifkovits *et al.*, 2014)	GSE54022	3	Mouse Gene v1.0 ST	Cardiac Fibroblasts (in vitro)	Stimulation with TGFβ 2 ng/ml vs control
TGFb MG	(Chodosh *et al.*, 2012)	GSE50968	3	Mouse Genome 430	Mammary Gland epithelial cells (in vitro)	Stimulation with TGFβ 15 ng for 6 hours vs control
Ret-Cer	(Su *et al.*, 2004)	GSE1133	2	GNF1M	whole tissue (in vivo)	Baseline expression retina vs cerebellum
Olf-Cer	(Su *et al.*, 2004)	GSE1133	2	GNF1M	whole tissue (in vivo)	Baseline expression olf. bulb vs cerebellum

Table S3. Overview of publicly available data sets used in the evaluation of LEAN; Rep.: Number of replicates per condition; olf.: olfactory

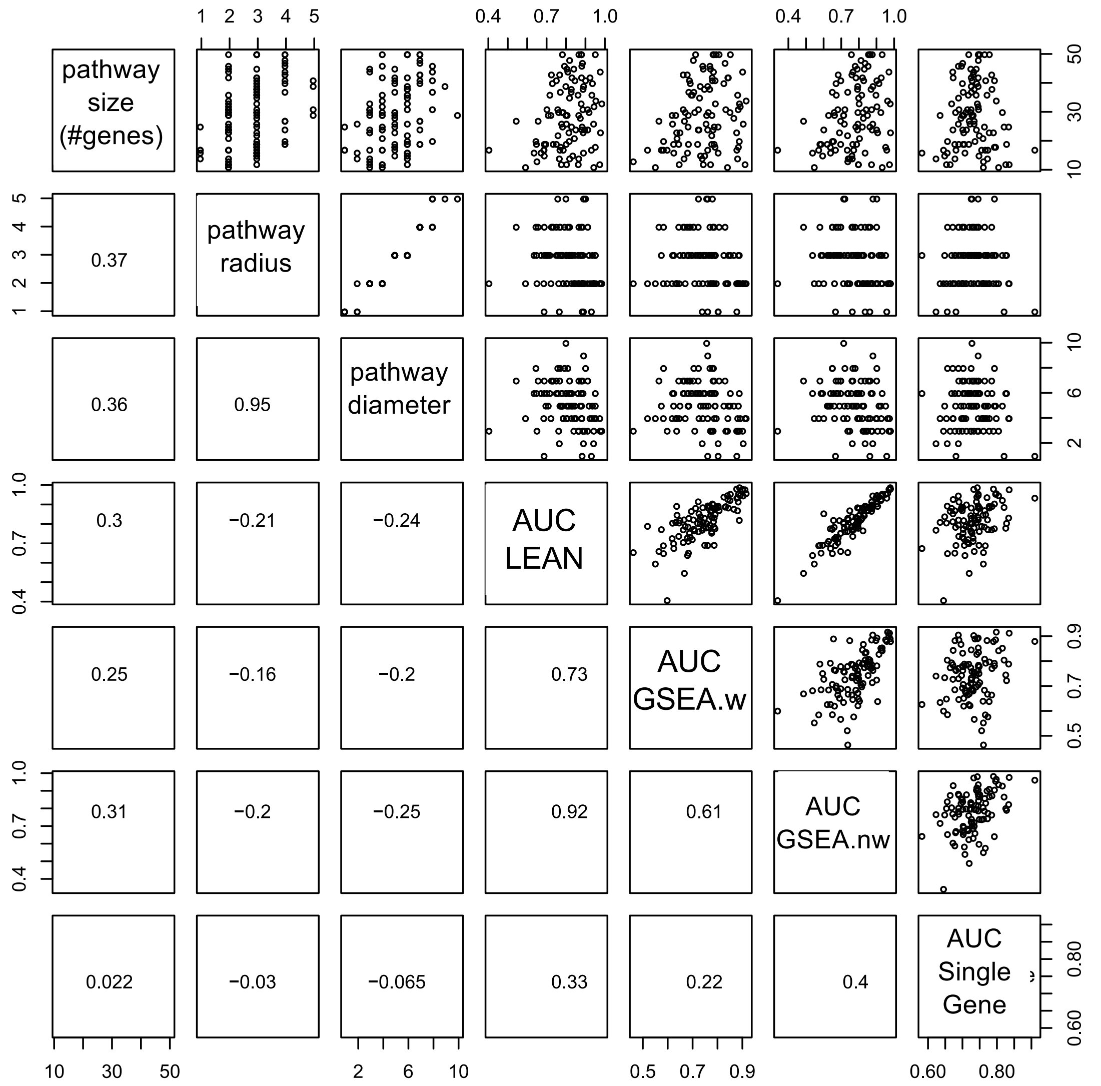

Fig. S1. Pathway simulation study: Dependency of LEAN ROC-performance on network statistics of the simulated subnetworks: The upper triangle of the matrix contains pair-wise plots showing dependencies between the ROC-performances (AUC) of LEAN as well as the GSEA enrichment statistic applied on the local subnetwork model and subnetwork characteristics like size (number of genes in subnetwork), graph radius and graph diameter for 100 separate subnetworks simulated as described in the Supplementary Methods section with p-values drawn using the parameter values $p_{\text{enr}} = 0.5$ and $p_{\text{scale}} = 0.1$. The lower triangle displays Pearson correlation between pairwise features.

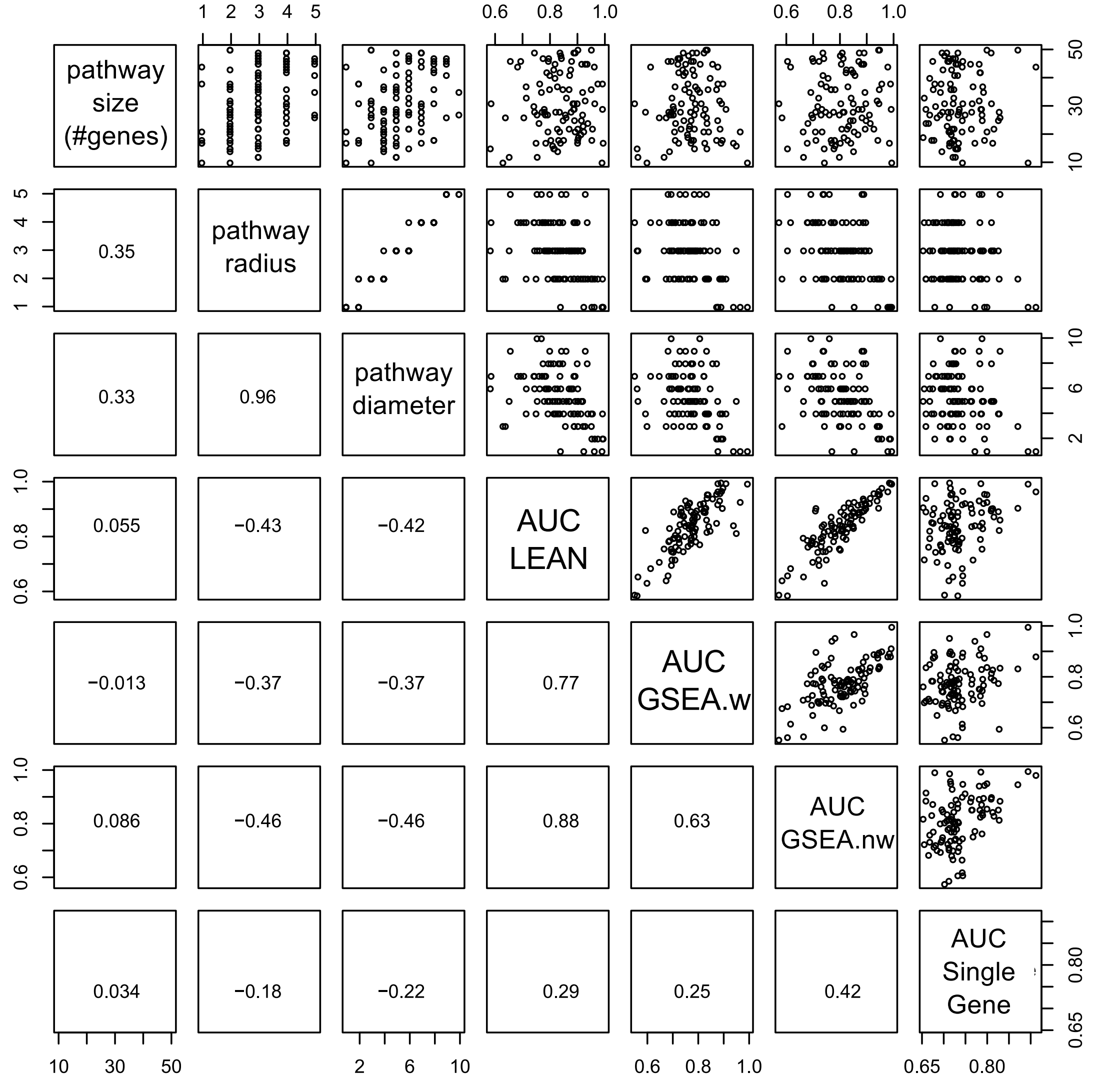

Fig. S2. Pathway simulation study: Dependency of LEAN ROC-performance on network statistics of subnetworks simulated without preferential attachment: The upper triangle of the matrix contains pair-wise plots showing dependencies between the ROC-performances (AUC) of LEAN as well as the GSEA enrichment statistic applied on the local subnetwork model and subnetwork characteristics like size (number of genes in subnetwork), graph radius and graph diameter for 100 separate subnetworks simulated as described in the Supplementary Methods section with p-values drawn using the parameter values $p_{\text{enr}} = 0.5$ and $p_{\text{scale}} = 0.1$. The lower triangle displays Pearson correlation between pairwise features.

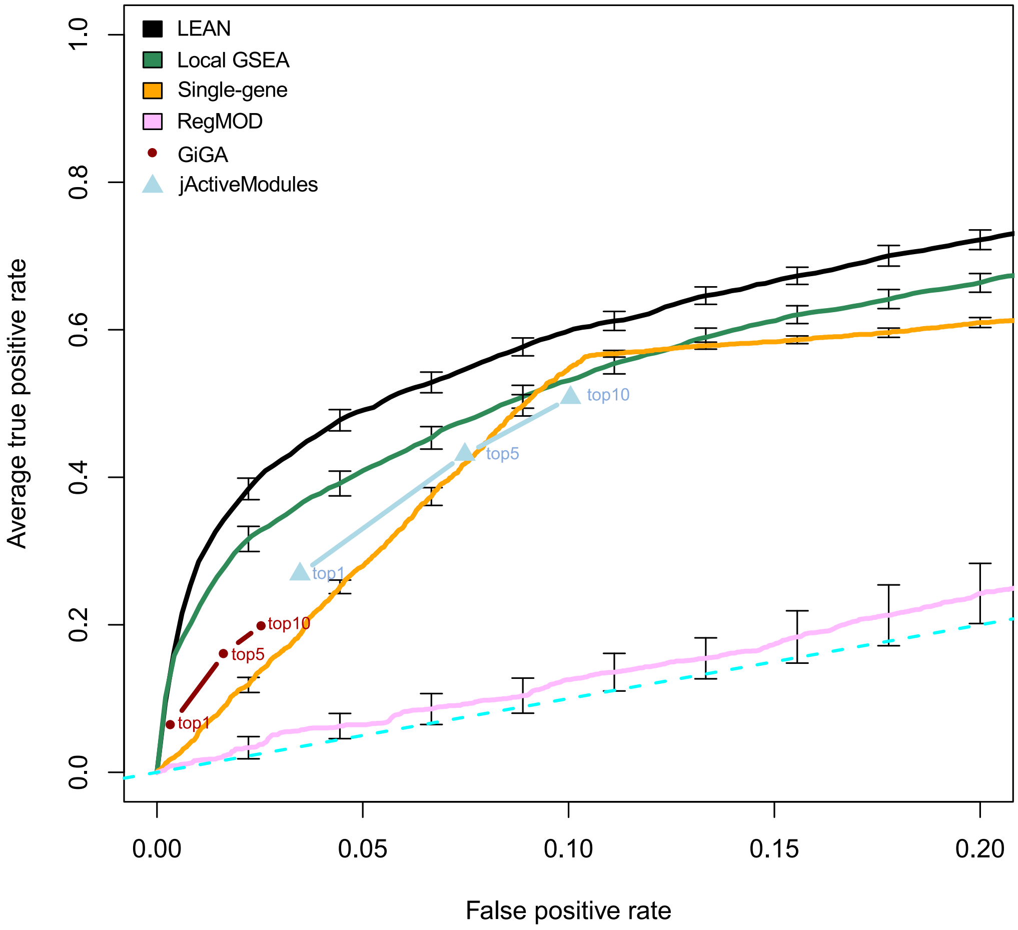

Fig. S3. ROC analysis results on subnetworks simulated without preferential attachment: The figure shows average true positive rates (TPR) over 10 separate pathway simulation instances at given false-positive rates (FPR). Error bars denote standard error of the mean. Average TPR and FPR obtained by the highest scoring, the five and the ten highest scoring jActiveModules and GiGA subnetworks, respectively, are shown. All pathway simulations used in the creation of this figure were run with $p_{\text{enr}} = 0.5$ and $p_{\text{scale}} = 0.1$ and equal probability of subnetwork extension disregarding node degrees.

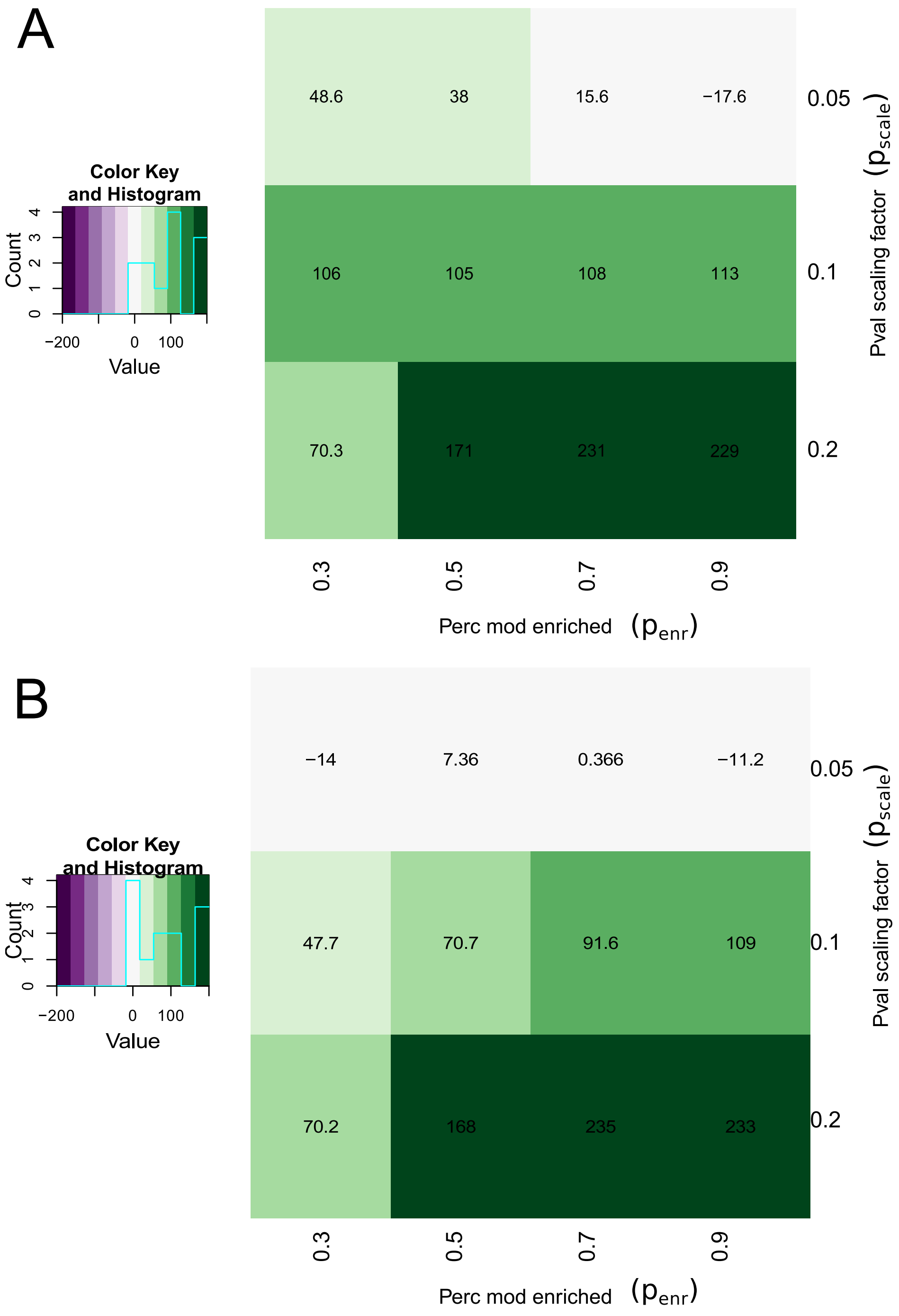

Fig. S4. Pathway simulation study: ROC analysis results of A) LEAN and B) Local GSEA expressed as percent improvement in terms of pAUC (FPR ≤ 0.05) over gene-by-gene scoring

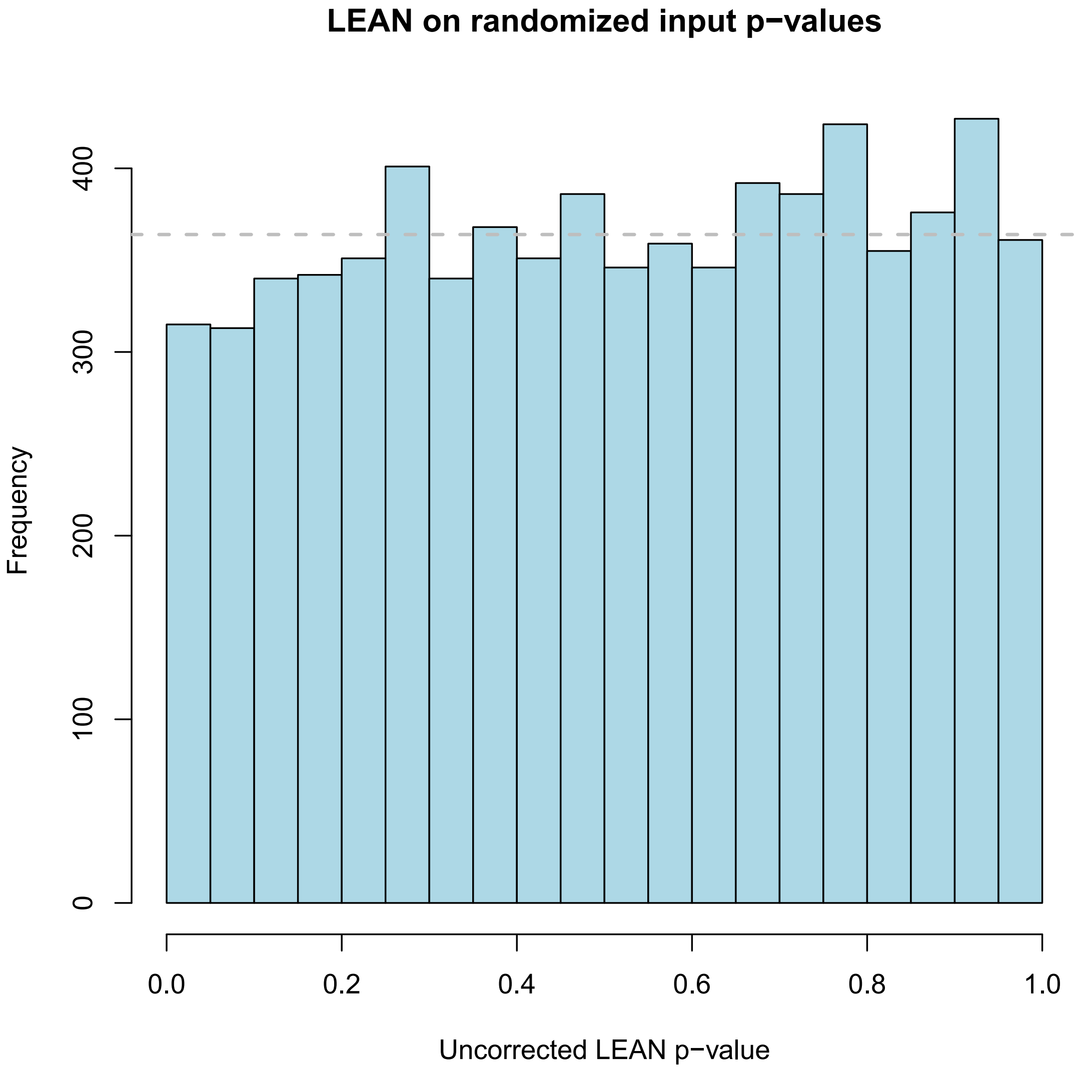

Fig. S5. Histogram of uncorrected LEAN p-values obtained when applying LEAN to the high-confidence STRING network and a randomly permuted version of the p-values obtained from the CCM2 experiment. The empirical p-value distribution approximates a perfect uniform distribution indicated by a grey dashed line.
