# Supplementary material for: Network-based analysis of omics data: the LEAN method
Source: Bioinformatics. 2016 Dec 6;33(5):701–9. doi: 10.1093/bioinformatics/btw676 (PMC5408824; doi:10.1093/bioinformatics/btw676)
Supplement: Supplementary Data [file btw676_supp.zip › LEANR_1.4.7.tar.gz!/LEANR/inst/doc/CCM-data.html]

LEAN on CCM data


# LEAN on CCM data

#### *Frederik Gwinner*

#### *2016-09-14*

# Run LEAN on the CCM data

Loading package and necessary data

```
library(LEANR)
set.seed(123456)
# load network and CCM p-values
data(g2)
data(CCM.pvals)
data(gene.annots)
```

Run LEAN on the precomputed differential expression p-values (computed using limma) in turn for each of the three CCM gene knock-out experiments. As we run these calculations with background distributions of size 10.000, they will take a little time depending on how many cores are available on your machine.

```
LEAN_results<-lapply(names(CCM.pvals),function(ccm){
  run.lean(CCM.pvals[[ccm]], g2, n_reps = 10000, ncores = 3)
})
names(LEAN_results)<-names(CCM.pvals)
```

Extract significant local subnetworks in each of the three experiments and show which local subnetworks are significant in all three knock-out experiments

```
# Extract significant local subnetworks
sign.genes<-lapply(LEAN_results,function(LEANres){
  rownames(LEANres$restab[LEANres$restab[,'PLEAN']<=0.05,])
})
names(sign.genes)<-names(CCM.pvals)

# Show local subnetworks detected as significant in all three knock-outs
all.sign<-intersect(intersect(sign.genes[[1]],sign.genes[[2]]),sign.genes[[3]])
print(gene.annots[all.sign,])
```

```
##                             ensembl_gene_id mgi_symbol entrezgene
## 10090.ENSMUSP00000107873 ENSMUSG00000001930        Vwf       <NA>
##                                                                              description
## 10090.ENSMUSP00000107873 Von Willebrand factor homolog [Source:MGI Symbol;Acc:MGI:98941]
```

The local subnetwork around VWF is the only one significant in all three knock-outs. Now we extract the local subnetwork around VWF and create a Cytoscape-readable subnetwork file

```
# Extract information on the detected local subnetwork (around VWF)
vwf.ls.info<-get.ls.info(all.sign[1],LEAN_results$CCM2)
print(head(vwf.ls.info))
write.table(vwf.ls.info,'./VWF_subnetwork_info_CCM2.txt',sep='\t',quote=F,row.names=F)

# Create sif file representing the local subnetwork around VWF
write.ls.to.sif(all.sign[1],LEAN_results$CCM2,'./VWF_subnetwork.sif')
```

After completing these steps, the local subnetwork around VWF can be inspected in Cytoscape by loading the network from file  and adding information about the contained genes by loading the table from file
