# Supplementary material for: Network-based analysis of omics data: the LEAN method
Source: Bioinformatics. 2016 Dec 6;33(5):701–9. doi: 10.1093/bioinformatics/btw676 (PMC5408824; doi:10.1093/bioinformatics/btw676)
Supplement: Supplementary Data [file btw676_supp.zip › LEANR_1.4.7.tar.gz!/LEANR/inst/doc/subnet-sim.html]

LEAN on simulated subnetworks


# LEAN on simulated subnetworks

#### *Frederik Gwinner*

#### *2016-09-14*

# Run LEAN on simulated subnetwork data

Loading necessary packages and data

```
library(LEANR)
library(ROCR)
set.seed(123456)
data(g2)
```

Create 10 instances of simulated subnetworks and gene p-values with default parameters: Number of modules = 10; Module size between 10 and 50; p\_scale = 0.1; p\_enrich = 0.5; with preferential attachment

```
n_instances=10

instances<-lapply (1:n_instances,function(i){
  subnet.simulation(g2, spec=sprintf('_STRING.fs900_rep%i',i), create.files=F)
})
```

Run LEAN on the subnetwork simulation instances. 1000 background permutations are enough for the purpose of the subnetwork simulation study. When we run these calculations for 10 separate subnetwork simulation instances, it still takes about 10 \* 1.5 = 15 minutes (using 3 cores).

```
LEAN_results_sim<-lapply(1:n_instances,function(i){
  pvals<-instances[[i]]$pvals[,'P.Value']
  names(pvals)<-rownames(instances[[i]]$pvals)
  run.lean.fromdata(pvals, g2, n_reps = 1000, ncores = 3)
})
```

Determine performance of LEAN and single-gene scoring on the subnetwork simulation data

```
# extract gene class labels for each of the simulation instances
gene.order<-rownames(instances[[1]]$pvals)
class_matrix<-t(foreach(i=1:length(instances),.combine=rbind) %do% {
  tmp<-!grepl('BG',instances[[i]]$pvals$NodeType);
  names(tmp)<-rownames(instances[[i]]$pvals);
  tmp[gene.order]
})

# extract LEAN scores for each of the simulation instances
LEAN_matrix<-t(foreach(i=1:length(instances),.combine=rbind) %do% {
  tmp<-LEAN_results_sim[[i]]$restab[,'pstar']
  names(tmp)<-rownames(LEAN_results_sim[[i]]$restab);
  tmp[gene.order]
})

# Extract single gene scores
SG_matrix<-t(foreach(i=1:length(instances),.combine=rbind) %do% {
  tmp<-instances[[i]]$pvals[,'P.Value']
  names(tmp)<-rownames(instances[[i]]$pvals);
  tmp[gene.order]
})

# ROC evaluation
predictions<-list(LEAN=prediction(-LEAN_matrix,class_matrix),
                  SG=prediction(-SG_matrix,class_matrix))

performances<-lapply(predictions,function(pred){performance(pred,"tpr","fpr")})
names(performances)<-names(predictions)

aucs_ind<-lapply(predictions,function(pred){performance(pred,"auc")@y.values})
aucs<-lapply(aucs_ind,function(x)mean(unlist(x)))
names(aucs)<-names(predictions)
```

Plot ROC curves

```
# plot resulting ROC curves
plot(performances$LEAN,xlim=c(0,.2),col='black',avg='vertical',lwd=3,
     spread.estimate='stderror',show.spread.at=seq(.02,.2,l=9),main='ROC performance on simulated subnetworks')
plot(performances$SG,col='orange',avg='vertical',lwd=3,add=T,
     spread.estimate='stderror',show.spread.at=seq(.02,.2,l=9))
abline(a=0,b=1,lty=2,col='cyan')
legend('topleft',c(sprintf('LEAN (AUC=%0.3g)',aucs$LEAN),sprintf('Single-gene (AUC=%0.3g)',aucs$SG)),fill=c('black','orange'))
```
